# Epimutational profile of hematologic malignancies as attractive target for new epigenetic therapies

**DOI:** 10.18632/oncotarget.10033

**Published:** 2016-06-14

**Authors:** Elisabetta Fratta, Barbara Montico, Aurora Rizzo, Francesca Colizzi, Luca Sigalotti, Riccardo Dolcetti

**Affiliations:** ^1^ Cancer Bio-Immunotherapy Unit, Centro di Riferimento Oncologico, IRCCS, National Cancer Institute, Aviano, PN, Italy; ^2^ University of Queensland Diamantina Institute, Translational Research Institute, Brisbane, Australia

**Keywords:** hematological malignancies, DNA methylation, histone modifications, azacytidine, 5-aza-2′-deoxycytidine

## Abstract

In recent years, recurrent somatic mutations in epigenetic regulators have been identified in patients with hematological malignancies. Furthermore, chromosomal translocations in which the fusion protein partners are themselves epigenetic regulators or where epigenetic regulators are recruited/targeted by oncogenic fusion proteins have also been described. Evidence has accumulated showing that “epigenetic drugs” are likely to provide clinical benefits in several hematological malignancies, granting their approval for the treatment of myelodysplastic syndromes and cutaneous T-cell lymphomas. A large number of pre-clinical and clinical trials evaluating epigenetic drugs alone or in combination therapies are ongoing. The aim of this review is to provide a comprehensive summary of known epigenetic alterations and of the current use of epigenetic drugs for the treatment of hematological malignancies.

## INTRODUCTION

**Figure 1:**
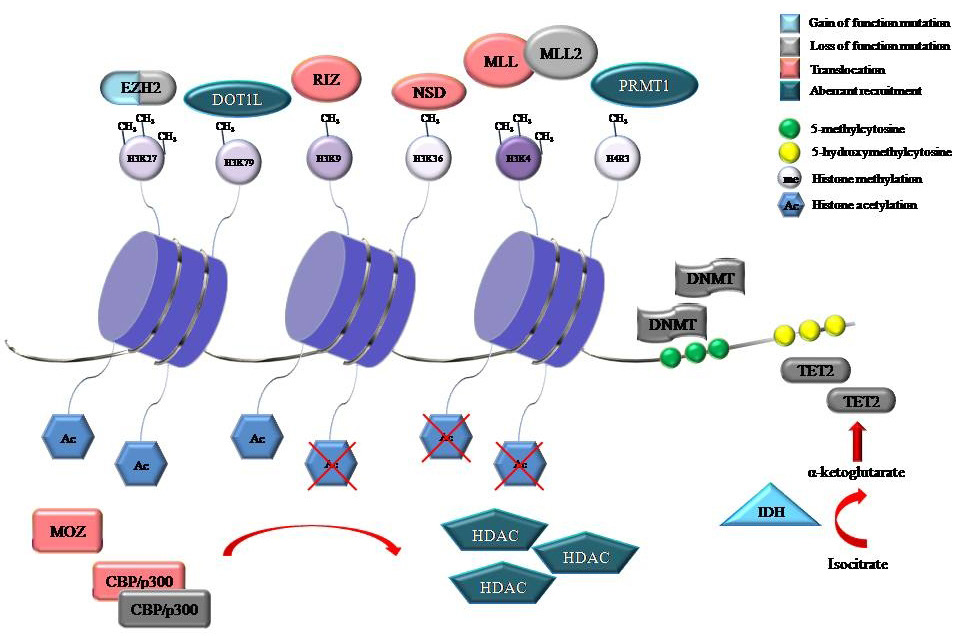
Representative proteins involved in DNA methylation and histone modifications that were identified to be recurrently mutated, translocated or aberrantly recruited in hematological malignancies The consequences of mutations, either gain of function or loss of function are also shown. Gene symbol: CBP, cyclic AMP response element-binding protein; DNMT, DNA methyltransferase; DOT1L, disruptor of telomeric silencing 1-like; EZH2, enhancer of zeste 2; H3K4, histone 3 lysine 4; H3K9, histone 3 lysine 9; H3K27, histone 3 lysine 27; H3K36, histone 3 lysine 36; H3K79, histone 3 lysine 79; H4R3, histone 4 arginine 3; HDAC, histone deacetylase; IDH, isocitrate dehydrogenase; MLL, mixed-lineage leukemia; MOZ, monocytic leukemia zinc finger protein; NSD, nuclear-receptor-binding SET-domain-containing; PRMT, protein arginine N-methyltransferase; RIZ, retinoblastoma protein-interacting zinc finger; TET2, Ten-Eleven-Translocation 2.

One of the main advances in understanding of cancer has been the observation that genetic changes to genes encoding for the epigenetic machinery are common and recurring events in oncogenesis and tumor progression. The application of next-generation sequencing technologies to tumor samples has allowed the identification of novel mutations and structural variations in proteins involved in DNA methylation and post-translational histone modifications, which are dynamically connected each other in the regulation of gene expression. Disruption of this complex epigenetic control mechanism has been frequently described in hematological malignancies, suggesting that alterations in epigenetic regulators may impair the expression of genes that regulate hematopoietic stem cells (HSC) proliferation, survival and stemness [1–4] (Figure 1).

In this review, we mainly focus on epigenetic alterations occurring in hematological malignancies and discuss the potential utility of epigenetic targeting in the treatment of these tumors.

## DNA METHYLATION

The best characterized epigenetic modification is DNA methylation, which occurs in the context of cytosine bases in the cytosine-guanine dinucleotides (CpG) in mammalian DNA strands. Cytosine modification is catalyzed by DNA methyltransferases (DNMTs), which transfer a methyl group from the donor S-adenosyl methionine (SAM) to the 5-carbon (C5) position of the pyrimidine ring. Transcriptional silencing by DNA promoter methylation has an essential regulatory function in several cellular processes, and is involved in establishing developmental tissue-specific patterns of gene expression. DNA methylation also plays a crucial role in establishing genomic imprinting and in maintaining balanced expression of X-linked genes in female cells through X chromosome inactivation. DNA methylation also safeguards genomic integrity and stability by silencing endogenous retroviral and parasitic repetitive sequences [5, 6]. Although five members of the DNMT family have been identified, only DNMT1, DNMT3A, and DNMT3B are known to be involved in the methylation of CpG sites. DNMT1 is classified as a maintenance DNMTs since it preferentially copies DNA methylation patterns from a hemi-methylated substrate after DNA replication. Conversely, DNMT3A and DNMT3B serve as *de novo* DNMTs that are essential in the generation of new methylation patterns during embryogenesis and germ-cell development. Additionally, there is a class of methyl-CpG-binding proteins (MBDs) which bind to methyl-CpG within gene promoters and prevent the binding of specific transcription factors to their recognition sites [7, 8].

Another family of important mediators of DNA methylation is the Ten-Eleven-Translocation (TET) family, which includes proteins that utilize oxygen and α-ketoglutarate (α-KG) to catalyze different reactions, including the oxidation of 5-methylcytosine (5-mC) to 5-hydroxymethylcytosine (5-hmC). The TET family of proteins was first identified as a fusion partner of mixed-lineage leukemia (MLL) in patients with t(10;11)(q22;q23) acute myeloid leukemia (AML). Although the biological functions of 5-hmC are still largely unknown, recent evidence suggests that it may play a functional role in stem cell biology [8].

DNA methylation appears to be critically involved in hematopoietic cell differentiation and the development of hematological malignancies, since several genes that regulate the processing of 5-mC are commonly found to be mutated in hematopoietic tumors [9].

### DNMTs

DNMTs are essential for early stage of hematopoiesis. The absence of DNMT-1 in HSC impaired self-renewal *in vitro*. Furthermore, mouse HSC lacking *Dnmt-1* were unable to suppress key myeloerythroid regulators and lost their ability to differentiate into lymphoid progeny, thus demonstrating that DNA methylation is necessary to protect normal HSC from lineage restriction [10]. More recently, Challen GA et al. demonstrated that *Dnmt3a* loss progressively impairs the differentiation capacity of HSC and is accompanied by a simultaneous expansion of HSC in the bone marrow. Furthermore, *Dnmt3a*-deficient HSC showed both hypermethylation and hypomethylation of promoter regions, resulting in an up-regulation of multipotency genes and a down-regulation of HSC-specific genes. Altogether, these data support the critical involvement of DNMT3A in regulating HSC differentiation [11] and suggest that the loss of *de novo* DNMT3A activity might impair the differentiation potential of HSC, providing a possible explanation for how DNMT3A mutations can contribute to AML and myelodysplastic syndrome (MDS) pathogenesis. In fact, several studies using large-scale array-based genomic resequencing and whole-genome sequencing of human leukemia have revealed recurrent DNMT3A mutations at multiple sites in AML patients. Greater than 50% of DNMT3A mutations occur at a single amino acid position, R882, located within the catalytic domain; this leads to reduced enzymatic activity *in vitro* [12]. Consistently, the presence of DNMT3A gene mutations was detected in approximately 20% of patients with AML, a genetic change associated with a shorter overall survival [13]. However, DNMT3A mutations did not correlate with any variations in 5-mC content in AML genomes and were not associated with a specific methylation or gene expression signature in AML patients, so further evaluation is needed to better define the potential pathogenic role of these mutations [13, 14].

### DNA hypermethylation

Several lines of evidence point to a role for DNA hypermethylation in the molecular pathogenesis of hematological malignancies (for review see [15]). In fact, the gene encoding the cell cycle regulator p15/INK4b is frequently inactivated by promoter hypermethylation in a large proportion of leukemia patients. Aberrant DNA hypermethylation impairs p15 growth-suppressive properties, allowing leukemic cells to escape inhibitory signals in the bone marrow. Hypermethylation of p15 promoter occurs in approximately 50% of patients with chronic myeloid leukemia (CML), AML, and acute lymphoblastic leukemia (ALL) and represents a key feature of the malignant progression of MDS [16]. In fact, increased CpG methylation at the INK4b locus was associated with the progression of MDS to AML, thus suggesting that aberrant p15 gene hypermethylation may be considered an early event in myeloid cell transformation [17]. A strict association between aberrant promoter methylation and DNMT expression has been found in MDS, a hematological malignancy in which the list of genes inactivated by hypermethylation has grown considerably (for review see [18]). Recently, using an *in vitro* MDS model, DNA hypermethylation of several genes involved in normal hematopoiesis was identified and associated with elevated DNMT isoform expression, supporting the notion that this disease is characterized by widespread epigenetic deregulation [19].

### DNA hypomethylation

Loss of methylation has been reported in several hematological malignancies. Genome-wide DNA methylation takes place predominantly at repetitive sequences, including short and long interspersed nuclear elements and LTR elements, segmental duplications and centromeric and subtelomeric regions [20, 21]. The Long Interspersed Nucleotide Element-1 (LINE-1) repetitive elements are the most well-documented interspersed repetitive elements displaying hypomethylation in various cancers, including ALL. Hypomethylation in the promoter region of LINE-1 can lead to the reactivation of transposable LINE-1 elements that may cause chromosomal instability, as observed in CML [22].

### TET enzymes and DNA hydroxymethylation

Mutations in TET2 have been found in a range of hematological malignancies, including AML, MDS, myeloproliferative neoplasms (MPN), and chronic myelomonocytic leukemia (CMML) with frequencies of 24%, 19%, 12% and 22%, respectively [23]. In a study involving patients with MDS and CMML, a TET2 loss-of-function mutation was detected in CD34+ cells, suggesting the early occurrence of this genetic change along the natural history of these malignancies [24]. It has been also reported that loss of Tet2 expression in *Tet2*-deficient mouse embryonic stem cells led to impaired hematopoietic differentiation, with expansion of HSC and multipotent progenitor cells. Particularly, *Tet2*-deficient mice developed hematopoietic malignancies resembling human CMML [25]. Altogether, these data might support the critical role for Tet2 in regulating 5-hmC levels within genes involved in the self-renewal, proliferation, and differentiation of HSC. As described above, TET2 function requires α-KG, a substrate produced from isocitrate by isocitrate dehydrogenase (IDH). Mutations affecting IDH1/2 were detected in up to 20 % of AML and consist of single amino acid substitutions occurring within the active site of the enzyme at one of three highly conserved arginine residues. All reported IDH1 and IDH2 mutations promote a new enzyme activity, with the acquisition of the ability to convert α-KG to produce 2-hydroxyglutarate (2-HG), which is similar in structure to α-KG, outcompetes α-KG for binding and inhibiting TET2 enzymes, thus altering gene expression and impairing lineage specific differentiation. Mutations of IDH1/2 were found to be mutually exclusive with TET2 mutations in a large cohort of AML patients. Consistent with a common role in AML pathogenesis, AML samples with mutations in either IDH1/2 or TET2 displayed overlapping DNA methylation signatures, characterized by global promoter hypermethylation [26].

## HISTONE MODIFICATION IN HEMATOLOGICAL MALIGNANCIES

Histones are abundant nuclear proteins involved in the formation of nucleosomes, structures upon which eukaryotic DNA is wrapped. Histones H2A, H2B, H3 and H4 can undergo several post-translational modifications mainly in their N-terminal tails, with methylation and acetylation being the most studied. These modifications, in turn, alter the electrostatic charge of histones and modify their binding to DNA, thus playing an essential role in determining the transcriptional status of chromatin, either inducing an open chromatin structure that is permissive for transcription or a more condensed chromatin state, which leads to transcriptional gene silencing. Modifications to histone tails can also create binding surfaces for protein recognition modules, such as bromodomains and chromodomains, which recruit specific functional complexes. So far, a wide range of possible combinations of histone modifications has been identified. Each histone tail can be either unmodified or modified, with lysine residues that can either be mono-, di-, or tri-methylated or acetylated. Based on this intricate pattern of histone modifications, Struhl and Allis proposed a “histone code” hypothesis, suggesting that non-histone proteins would be able to “write”, “read” and “erase” histone modifications and/or their combinations in order to regulate specific chromatin functions and, therefore, to determine the transcriptional status of the target gene (reviewed in [27]). In all higher eukaryotes, canonical histones are encoded by multiple gene copies, giving rise to several variants with different sequences [28]. The “canonical” histones are expressed at high levels during the S-phase of the cell cycle, allowing for their rapid deposition behind the DNA replication fork. By contrast, replication-independent histone “variants” are expressed and incorporated into chromatin throughout the cell cycle (for reviews see [29]). Canonical histone genes are located within multigene clusters, and their expression is largely regulated by a unique 3′ end mRNA which is not polyadenylated, but instead contains a 26 bp sequence that forms a stem-loop structure. On the contrary, genes for histone variants are typically found in a single or low copy number, and are regulated similarly to normal genes [30]. The incorporation of histone variant into nucleosomes may be influenced by DNA methylation. A handful of studies in both plants and mammals report that the H2A variant H2A. Z is excluded from methylated DNA at promoters and within gene bodies, suggesting that epigenetic factors also contribute to histone localization [31, 32].

Mutations affecting genes encoding for canonical histones and their variants have an important role in altering chromatin architecture, leading to aberrant gene expression. Mutations in H3F3A, which encodes the histone H3 variant H3.3, have been recognized as being especially important in promoting aberrant global histone modification changes in pediatric glioblastoma. However, H3F3A mutations represent a very rare event in hematopoietic tumors, since they occur only in a ALL with a very low incidence [33]. In addition to H3F3A, the H2AFX gene also seems to be involved in both cancer initiation and progression. Deletion of band 11q23, where H2AFX maps, has been detected in several human cancers, including T cell prolymphocytic leukemia and B-CLL [34]. Furthermore, a G/A single nucleotide polymorphisms upstream of the start codon of H2AFX has been associated with non-Hodgkin lymphoma (NHL) susceptibility and development [35]. Histone gene mutations are not restricted to histone H2 and H3, since mutations affecting H1 family members have also been observed in follicular lymphoma [36]. H1 gene mutations are located within the H1 globular domain and result in reduced capacity to associate with chromatin [37] and binding to DNMT3B [38]. However, it is not yet clear if histone H1 mutations lead to defective chromatin compaction or result in chromosomal instability.

### Histone methylation

Histone methylation involves the addition of methyl groups to the side chain nitrogen atoms of arginine, lysine and histidines residues, with the most commonly histone methylation sites including histone H3 lysine 4 (H3K4), H3K9, H3K27, H3K36, H3K79 and H4K20. Depending on which residue is modified, histone methylation is associated with both transcriptional gene activation and repression. The pattern of histone methylation is read by histone methyltransferases (HMTs) that catalyze the addition of methyl groups donated from S-adenosylmethionine to histones. So far, three families of HMTs have been identified: the SET-domain-containing proteins and disruptor of telomeric silencing 1-like (DOT1L) proteins, which methylate lysines, and members of the protein arginine N-methyltransferase (PRMT) family, which methylate arginines (reviewed in [39]).

The EZ homolog 2 (EZH2) protein contains a SET domain that is responsible for the trimethylation of H3K27, a histone modification associated with transcriptional silencing [40]. The EZH2 gene is highly expressed in germinal center B (GCB) cells, indicating it has a role in normal GCB cellular physiology [41]. As B cells exit the GCB, EZH2 expression usually decreases, allowing the expression of genes that are involved in terminal differentiation [42]. A recurrent mutation of tyrosine 641 (Y641) within the EZH2 SET domain has been detected in approximately 22% of diffuse large B-cell lymphoma (DLBCL) and 7% of follicular lymphomas (FL) [43]. This mutation was the first of chromatin-modifying gene alteration to be described in DLBCL and FL. Surprisingly, lymphoma cell lines carrying the Y641 mutation showed increased H3K27 trimethylation. In light of these observations, *in vitro* experiments demonstrated that the mutated EZH2 form was defective in catalyzing H3K27 monomethylation, but acquired enhanced catalytic efficiency for the H3K27 trimethylation [44, 45]. A global increase in H3K27 trimethylation, due to EZH2 Y641 mutation, represses genes involved in proliferation checkpoints and associated with B-cell differentiation, thus providing a possible explanation for the oncogenic role of mutant EZH2 in DLBCL [41, 46]. A series of loss-of-function EZH2 mutations have been identified in patients with myeloid malignancies, such as MDS, CMML, and primary myelofibrosis [47–49]. Although these studies demonstrated that EZH2 loss resulted in a global decrease in H3K27 trimethylation, the molecular mechanism by which the inactivation of EZH2 contributes to hematopoietic transformation is yet to be determined.

The nuclear-receptor-binding SET-domain-containing (NSD) family consists of three members, NSD1, NSD2, and NSD3, which preferentially target H3K36 methylation. Although NSD proteins are frequently found to be translocated in leukemia, little is known about their role in transcriptional regulation. In childhood AML, the NSD1 gene is disrupted by the t(5,11)(q35;p15.5) chromosomal translocation, which produces the fusion protein NUP98–NSD1 [50]. *In vitro* studies have indicated that the chimeric protein NUP98-NSD1 promotes the transcription of the Homeodomain (HOX) genes [51], which are normally involved in proliferation and differentiation of HSC. This epigenetic deregulation of HOX genes sustains the self-renewal of myeloid stem cells that is essential for the development of AML. A second gene of the family, NSD2, may fuse with the IgH locus via t(4;14) translocation, which affects 15-20% of patients with multiple myeloma (MM) [52]. Chromosomal fusion leads to overexpression of the NSD2 protein, which may promote the proliferation of MM cells presumably by reprogramming global histone methylation and gene expression [53].

MLL is a SET domain family protein that plays a role in H3K4 methylation. The first gene of this family, MLL, was shown to target SET domain HMT activity to positively regulate HOX genes transcription in hematopoiesis through di- and tri-methylation of H3K4 at HOX promoter and enhancer sequences [54]. Rearrangements affecting the MLL gene are normally found in approximately 10% of AMLs in adults and in more than 70% of infant leukemia [46]. Chromosomal rearrangements involving MLL can occur in several forms such as balanced translocations, inversions, and partial tandem duplications of 11q23. Balanced translocations are the most common rearrangement disrupting MLL and more than 50 different partner genes are known to date [55–57]. Although some of these translocations delete the C-terminal containing the SET domain, and therefore HMT activity [58], the resultant MLL fusion proteins seem to act as dominant positive transcriptional regulators capable of transforming both HSC and early myeloid progenitors. Recent evidence indicates that MLL fusion proteins may be involved in the regulation of gene expression by directing histone modification. The most common MLL fusion proteins, including MLL-AF4, MLL-AF9, and MLL-ENL, belong to a set of multiprotein complexes involved in transcriptional activation/elongation. These complexes can recruit DOT1L, an HMT that lacks the canonical SET domain and is responsible for catalyzing the methylation of H3K79. Studies on different human MLL-rearranged leukemia cells have demonstrated high levels of H3K79 methylation at HOX genes and other MLL targets, thus suggesting that aberrant H3K79 methylation may represent a mechanism of oncogenic transcriptional activation in MLL leukemia [46, 59]. MLL fusion proteins can also transform primary myeloid progenitors by directing the histone arginine methyltransferase PRMT1 to the promoter regions of HOX genes, which become aberrantly methylated at H4 arginine 3 and actively transcribed [60]. Translocations involving MLL has been widely described in the last years, but loss of function mutation affecting MLL2 have also been found in a significant percentage of malignancies, including NHL. In particular, in 89% of FL and 32% of DLBCL, MLL2 mutations resulted in small deletions creating frameshifts or point mutations that introduced premature stop codons in the catalytically active SET domain [43, 61, 62]. However, the effect of MLL2 mutations on H3K4 methylation and the mechanism by which MLL2 loss contributes to lymphomagenesis are still unclear. Another member of this family, MLL3, has been found mutated in FL and DLBCL [63, 64], suggesting a functional redundancy in oncogenic transformation.

The retinoblastoma protein-interacting zinc finger (RIZ) is a HMT involved in H3K9 methylation [65], which is associated with transcriptional silencing. RIZ proteins contain a domain, called the PR domain, showing similarity with the SET domain. The RIZ gene produces two related proteins, RIZ1 and RIZ2, which differ at the N-terminal domain by the presence or absence of the PR domain, respectively. The RIZ1 product commonly undergoes deletions in several types of human cancers. Consistently, mice lacking RIZ1, but not RIZ2, were found to develop unusual tumors, such as diffuse large B-cell lymphoma, suggesting that the PR domain is likely responsible for the tumor suppressing activity of RIZ1 [66]. Another member of the of the RIZ family is the MDS1-EVI1 gene, which maps to the 3q26 locus and encodes two products with different lengths: MSD1-EVI1, containing the PR domain with HMT activity, and EVI1, lacking the PR domain [67]. The PR domain-containing product is known to possess tumor suppressor functions, while EVI1 has a positive role in oncogenesis. MSD1-EVI1 and EVI1 are expressed in hematopoietic cells and their inappropriate expression has been implicated in the development of myeloid disorders. Rearrangements such as translocations or inversions involving the 3q26 band usually cause disruption of the MDS1-EVI1 protein and overexpression of EVI1, as observed in AML and MDS [68, 69]. However, the mechanism by which EVI1 overexpression contributes to oncogenic transformation remains unclear.

### Histone acetylation

Acetylation of the ε-amino group of lysine residues is an important post-translational modification that occurs not only in histone tails, but also in chromatin proteins and non-histone proteins regulating important pathways. Acetylation of lysine residues neutralizes the charge on histones, leading to an open chromatin structure that is more permissive for gene transcription, whereas deacetylation of lysines promotes chromatin condensation and transcriptional gene silencing. The dynamic equilibrium of lysine acetylation *in vivo* depends on the balance of the activities of histone acetyl transferases (HATs) and HDACs [70].

#### HATs

According to their homology, known HATs can be grouped into different families, including the MYST superfamily, the GCN5 *N*-acetyltransferase (GNAT) family, and the cyclic AMP response element-binding protein (CBP)/p300 superfamily [70].

MOZ, a member of the MYST family, has been found to acetylate both itself and lysine (K) residues on H2B, H3 and H4 *in vitro* and H3K9 *in vivo*. Besides its intrinsic HAT activity, MOZ functions as co-activator of several transcription factors that are involved in the proliferation and differentiation of HSC [71]. Recurrent reciprocal translocations fuse the MOZ gene to genes encoding CBP and p300 or the protein transcription intermediary factor 2 (TIF2). Fusion proteins resulting from MOZ chromosomal translocation can transform hematopoietic progenitors *in vitro* and induce myeloproliferative disease *in vivo* [72]. The chimeric protein MOZ-TIF2, which derives from the inversion inv8(p11q13), retains the conserved HAT domain of MOZ and the CBP-interaction domain of TIF2. In one of the earliest studies, Deguchi et al. reported that the expression of MOZ-TIF2 immortalized myeloid progenitors *in vitro* and induced AML in mice [73]. Further studies in AML demonstrated that MOZ-TIF2 reduced CBP activity *in vivo*, resulting in aberrant acetylation patterns that inhibited transcriptional activities of critical cell cycle regulators, such as p53 [74].

Similarly to MOZ, CBP is also involved in HSC self-renewal by regulating the activity of transcriptional complexes. Heterozygous loss of CBP impairs HSC self-renewal and predisposes to hematologic malignancies in humans and in mouse models [75]. Consistently, it has been shown that CBP plays an important role in tumorigenesis through its ability to modulate the transcriptional activity of several transcription factors, such as NF-κB and p53 [76]. The CBP gene is located on chromosome 16p13, which is target of translocations in AML and MDS, including MOZ-CBP [t(8;16)(p11;p13)]. This fusion gene encodes for a protein that retains most of the interaction domains of both parental proteins as well as the HAT domain of MOZ. The resulting MOZ-CBP fusion protein has been found to interact with the p65 subunit of NF-κB and to increase the expression of NF-κB target promoters in HSC from individuals with AML [77]. Besides the translocations mentioned above, inactivating mutations and deletions in the HAT coding domain of CBP and its paralog p300 have been detected in approximately 41% of FL and 39% of DLBCL. The majority of these mutations affected only one allele, suggesting that reduced HAT activity may have a critical role in lymphomagenesis. In fact, CBP/p300 genetic lesions impair their ability to acetylate known substrates such as BCL6 and p53, that are themselves targeted by somatic mutation in DLBCL [62]. Reduced p53 tumor suppressor function and constitutive activation of BCL6 oncoprotein may represent alternative oncogenic mechanisms by which CBP/p300 mutations contribute to DLBCL transformation.

#### HDACs

So far, mammalian HDACs have been divided into four major classes, based on their sequence similarities to yeast HDACs, subunit localizations, and enzymatic activities: class I (HDAC 1, 2, 3, and 8), class II (HDAC 4, 5, 6, 7, 9, and 10), class III or sirtuins (SIRT 1-7) and class IV which consists of HDAC 11 [78]. Class I HDACs, which share homology with the RPD3 yeast protein, are primarily localized in the nucleus, and are ubiquitously expressed in several human cell lines and tissues. Class II HDACs are homologues to the Hda1 yeast protein and are expressed in a limited number of cell types. They are mainly cytoplasmic, but also shuttle between the nucleus and cytoplasm [79]. Sirtuin members, which are homologues to the Sir2 yeast protein, have been found in a wide variety of subcellular locations [80]. Class IV HDACs share sequence similarity with the catalytic core of both Class I and II enzymes and are found predominantly in the cytoplasm where they preferentially deacetylate non-histone proteins [81].

The first evidence of the involvment of HDACs in oncogenesis was derived from hematological malignancies, where fusion proteins resulting from chromosomal translocations were shown to aberrantly recruit HDACs to specific gene promoters, thereby impairing the differentiation and proliferation of myeloid cells [82]. In this respect, acute promyelocytic leukemia (APL) represents a well described model, in which the retinoic acid (RA) pathway is disrupted and the myeloid differentiation is arrested at the promyelocytic stage [83]. RA receptor (RAR)-α is important for myeloid differentiation and acts as a transcriptional regulator by binding its partner RXR. The heterodimerization process enhances RAR-α binding affinity to RA response elements (RAREs) within the promoters of target genes, thus increasing its transcriptional efficiency [81]. In the absence of RA, the RAR-α-RXR heterodimer recruits HDAC-containing repression complexes that lead to chromatin condensation and transcription repression. Physiological concentrations of RA induce a conformational change in the RAR-α-RXR complex, resulting in the dissociation of repression complexes and the subsequent recruitment of transcription factors [84, 85]. In 100% of APL cases, the RAR-α gene is involved in reciprocal chromosomal translocations and, in >90% of these cases, the translocation partner is promyelocytic leukemia protein (PML), resulting in the formation of the PML-RAR-α chimeric protein, which acts as a transcriptional repressor. The PML-RAR-α fusion protein shows an increased ability to bind to DNA at RAREs and to associate with corepressor complexes containing HDACs and chromatin-modifying factors, such as HMTs and DNMTs that mediate the establishment of a repressive chromatin structure at RARα target promoters [86, 87]. PML-RAR-α fusion proteins do not dissociate from corepressor complexes at physiological concentrations of RA, resulting in the transcriptional silencing of RAR-α target genes which normally play an important role in the control of myeloid cell differentiation. Furthermore, PML-RAR-α fusion proteins have been shown to bind to non-canonical RAREs, resulting in widespread transcriptional deregulation that strongly interferes with gene expression programs involved in myeloid proliferation and hematopoietic progenitor self-renewal [88, 89].

Similar mechanisms of transcriptional disruption via histone deacetylation have been described in AML, where the chimeric AML1/ETO gene is produced by the fusion of the AML1 gene on chromosome 21 to the Eight-Twenty One (ETO) oncogene on chromosome 8. The t(8;21) was the first translocation to be discovered and represents one of the most frequent chromosomal abnormalities in AML [90]. Although AML1 functions as a transcriptional activator through the interaction with a complex containing p300, in the AML1-ETO fusion protein the HAT-interacting region of AML1 is replaced by the coding sequence of ETO. The resulting aberrant fusion protein has multiple effects on the regulation of proliferation, differentiation, and viability of leukemic cells by recruiting enzymes involved in epigenetic regulation. In fact, the ETO transcription factor interacts with repressor complex containing HDAC, but also associates with DNMT1, thus imposing aberrant transcriptional repression through intensive histone deacetylation and DNA hypermethylation at AML1 target hematopoietic promoters [88].

### Bromodomain proteins

Bromodomains (BRDs) represent highly evolutionarily conserved protein-interaction modulesthat recognize acetylated lysine residues in histones and other non-histone proteins. To date, over 40 nuclear or cytoplasmic BDRs have been identified, with each protein containing as many as six bromodomains. Human BRDs belong to eight distinct families and include chromatin-modifying enzymes, chromatin remodellers, transcriptional co-activators and mediators, and the large group of bromodomain and extra-terminal motif (BET) proteins. BET proteins utilize dual, tandem N-terminal BRD modules (BD1 and BD2) able to bind to acetylated lysine residues preferentially on H3 and H4 histone tails and on non-histone targets such as the NF-κB subunit RelA and GATA1 [91, 92]. The BET family consists of four members that regulate transcription by RNA polymerase II (Pol II): BRD2, BRD3, BRD4, which are ubiquitously expressed, and BRDT, the expression of which is restricted to germ cells. BET proteins do not possess enzymatic activity at chromatin, but function as scaffolds for a number of transcription factors or chromatin-modifying enzymes (for reviews see [93, 94]). Novel global discovery proteomics have recently identified BET protein as obligate components of core transcriptional regulatory machineries, including the polymerase-associated factor complex (PAFc) and the super elongation complex (SEC), which are critical regulator of MLL translocated leukemias [95]. Furthermore, BET proteins have been found to associate with a great number of active promoters and to converge upon a significant fraction of active enhancers of key genes, including critical oncogenic targets such as c-MYC [96–98]. This suggests a role for BET proteins as epigenetic readers and modifiers in leukemias and other hematologic malignancies.

## EPIGENETIC THERAPIES IN HEMATOLOGICAL MALIGNANCIES

As described above, enzymes that maintain and modify the epigenome play a crucial role in the regulation of normal hematopoiesis and, as such, they are often targeted by somatic alterations in hematological malignancies. In this context, epigenetic drugs are considered as an important therapeutic modality for the clinical management of hematological malignancies. Epigenetic drugs can be used alone or in combination therapies with currently available cancer chemotherapies.

### DNMT inhibitors

So far, the most widely studied DNMT inhibitors (DNMTi) 5-azacytidine (azacitidine, Vidaza) and 5-aza-2′-deoxycytidine (5-AZA-CdR, Decitabine, Dacogen) have undergone intensive clinical development that led to their Food and Drug Administration (FDA) approval for patients affected by MDS [99]. Once taken up by the cell, the nucleoside analogs azacitidine and 5-AZA-CdR undergo a series of metabolic modifications prior to being incorporated into genomic DNA during the S phase of the cell cycle. After incorporation, they behave as suicide substrates for the DNMTs that, in the attempt to methylate them, became irreversibly inactivated through the formation of a stable covalent bond between the enzyme and the modified pyrimidine ring. This binding leads to depletion of DNMT1 and passive hypomethylation of the genome following DNA replication. Azacitidine incorporates into both RNA and DNA, thus inhibiting protein synthesis, while 5-AZA-CdR is incorporated only into DNA [100]. Azacitidine and 5-AZA-CdR have been employed to treat hematological malignancies for a long time using different doses and schedules, alone or in combination with inhibitors of HDAC (for reviews see [101, 102]) (Table 1).

**Table 1:** Summary of clinical trials examining DNA hypomethylating agents (DHA) alone or in combination with Histone Deacetylase inhibitors in hematological malignancies

DHA	Phase	Combination	Hematological disease	Identifier	State
Azacitidine	I	Entinostat	AML^a^, CMML, MDS,	NCT00101179^b^	Ongoing
			MDS, AML, CMML	NCT00528983	Ongoing
		VPA, ATRA	AML, MDS	NCT01575691	Completed
			AML, MDS	NCT00350818	Completed
		Belinostat	AHM	NCT00351975	Completed
			MDS	NCT01152346	Completed
			AML, CMML, MDS	NCT01519011	Completed
		Pracinostat	AHM, MDS	NCT00741234	Completed
			MDS	NCT01571648	Completed
		Panobinostat	AML, MDS, CMML	NCT01613976	Completed
		Sodium phenylbutyrate	AML, MDS	NCT00004871	Completed
			AML, MDS, CMML, HL, MM, NHL	NCT01908387	Terminated
	I/II	Mocetinostat	MDS	NCT02018926	Recruiting
		Romidepsin	HL, NHL	NCT01998035	Recruiting
		Vorinostat	AML, DMS	NCT00392353	Ongoing
			MDS	NCT01305460	Ongoing
			AML, MDS	NCT01835587	Ongoing
		Vorinostat, Gemtuzumab ogamicin	AML	NCT00895934	Completed
		Mocetinostat	AML, MDS	NCT00324220	Completed
	II		MDS, JMML	NCT02447666	Recruiting
			MDS	NCT01652781	Recruiting
			AML, MDS	NCT02204020	Recruiting
			CMML, MDS	NCT01404741	Recruiting
			AML	NCT02450877	Recruiting
		Vorinostat	AML, MDS	NCT01617226	Recruiting
		Panobinostat	AML, CMML, MDS	NCT00946647	Recruiting
		VPA, Lenalidomide	MDS	NCT01342692	Recruiting
		VPA	AML, MDS	NCT02124174	Recruiting
			AML, MDS	NCT01995578	Recruiting
			AML, MDS	NCT01462578	Recruiting
			MDS	NCT02281084	Recruiting
		Vorinostat	AML, MDS	NCT00948064	Ongoing
		Entinostat	AML, CMML, MDS,	NCT00313586	Ongoing
			MDS	NCT01599325	Ongoing
		Pracinostat	MDS	NCT01873703	Ongoing
			MDS	NCT00721214	Ongoing
		VPA, ATRA	AML, MDS	NCT00326170	Completed
		VPA, Cytarabine	AML, DMS	NCT00382590	Completed
			MDS	NCT00897130	Completed
			AML	NCT00739388	Completed
			AML	NCT00387647	Completed
			MDS	NCT00384956	Completed
		VPA, ATRA	MDS, AML	NCT00339196	Completed
		Sodium phenylbutyrate	AML, MDS, MM, NHL	NCT00006019	Completed
		VPA, ATRA	MDS	NCT00439673	Completed
			MDS	NCT00102687	Completed
			AML, MDS	NCT00915785	Completed
			CMML	NCT01235117	Completed
			MDS	NCT00446303	Terminated
			CLL	NCT00413478	Terminated
		Mocetinostat	AML, MDS	NCT00666497	Terminated
		Mocetinostat	HL, NHL	NCT00543582	Terminated
	II/III	Lenalidomide, Vorinostat	CMML, MDS	NCT01522976	Ongoing
	III		AML, MDS	NCT00887068	Recruiting
		CCR	AML	NCT01074047	Ongoing
			MDS	NCT01186939	Completed
			MDS	NCT00071799	Completed
			AML, MDS	NCT00422890	Completed
	IV		MDS	NCT01201811	Completed
Decitabine	I	OSU-HDAC42	AML	NCT01798901	Ongoing
		Vorinostat	AML, CMML, MDS	NCT00357708	Completed
		Vorinostat	AML, ALL, CLL, NHL	NCT00275080	Completed
		VPA	NHL	NCT00109824	Completed
		VPA	AML, CLL, SLL	NCT00079378	Completed
			AML, ALL	NCT00042796	Terminated
		Romidepsin	MDS	NCT00114257	Completed
		Cytarabine, Vorinostat	AML	NCT01130506	Completed
			MDS	NCT00941109	Completed
			MDS	NCT00796003	Completed
			ALL	NCT00349596	Completed
			AML, MDS	NCT00049582	Terminated
	I/II	Panobinostat	AML, MDS	NCT00691938	Ongoing
			MDS	NCT01165996	Completed
			MDS,	NCT00075010	Completed
	II		AML, MDS	NCT01687400	Recruiting
		VPA, ATRA	AML	NCT00867672	Recruiting
		VPA	AML, MDS	NCT00414310	Completed
			CMML, MDS	NCT00067808	Completed
			MDS	NCT00003361	Completed
			AML	NCT00866073	Completed
			MDS	NCT00619099	Completed
			CML	NCT00042003	Completed
			CML	NCT00042016	Completed
			CML	NCT00041990	Completed
			MDS	NCT00744757	Completed
			CMML, MDS	NCT00113321	Terminated
			MDS	NCT01333449	Terminated
	III		MDS	NCT01751867	Completed
			MDS	NCT00043381	Completed
Azacitidine versus Decitabine	II		MDS	NCT02269280	Recruiting
	II		MDS	NCT01720225	Recruiting
	III		MDS	NCT01409070	Completed
	IV		MDS	NCT01011283	Terminated
SGI-110	I		AML	NCT02293993	Recruiting
	I/II		AML, CMML, MDS	NCT01261312	Ongoing
	II	Idarubicin, Cladribine	AML	NCT02096055	Recruiting
			MDS	NCT02131597	Recruiting
			MDS	NCT02197676	Recruiting
	III		AML	NCT02348489	Recruiting

a AHM, advanced hematologic cancers; AML, acute myeloid leukemia; ATRA, all-trans retinoic acid; CCR, Conventional Care Regimen; CLL, chronic lymphocytic leukemia; CML, chronic myeloid leukemia; CMML, chronic myelomonocytic leukemia; HL, Hodgkin's lymphoma; JMML, juvenile myelomonocytic leukemia; MDS, myelodysplastic sindrome; MM, multiple myeloma; NHL, non-Hodgkin lymphoma; SLL, small lymphocytic lymphoma; VA, valproic acid.

b Identifier of the trial as retrieved April, 2016, from: http://clinicaltrials.gov.

Initially, these drugs were used at relatively high doses, which however turned out to be toxic and did not show a satisfactory antitumor activity. By contrast, lower doses of azacitidine and 5-AZA-CdR resulted in a stronger DNA hypomethylating activity and a better antineoplastic effect [103].

The initial trials that demonstrated the efficacy of azacitidine started in 1984 and were carried out by the Cancer and Leukemia Group B (CALGB). In the first phase II study, named CALGB 8421, 48 patients with MDS were enrolled and treated with monthly azacitidine, 75 mg/m^2^/day continuous infusion for 7 days. Complete response (CR) was seen in 15% of azacitidine -treated patients, partial remission (PR) was rare, and 21% of patients showed hematological improvement (HI) [104]. Similar response rates were observed in the subsequent CALGB 8921 trial that included 70 patients with MDS, treated with either intravenous or subcutaneous azacitidine (75 mg/m^2^/d for 7 days every 28 days) [104]. In the last phase III randomized clinical trial CALGB 9221, a total of 191 patients with MDS were randomized to receive azacitidine 75 mg/m^2^/day subcutaneously for 7 days every 28 days, or supportive care. The results demonstrated a 60% overall response for MDS patients in the azacitidine arm compared to those treated with supportive care only. In particular, in MDS patients treated with low-dose azacitidine, CR, PR and HI were documented in 7%, 16% and 37% of cases, respectively. Furthermore, the median time-to-progression to AML or death was 13 months for patients in the supportive care arm compared with 21 months for patients randomly assigned to azacitidine, with significantly improved quality of life [104]. These results led to azacitidine approval by the FDA for the treatment of MDS. These three CALGB trials included a large number of patients with refractory anemia with excess blasts in transformation that, according to World Health Organization (WHO) criteria, was reclassified as AML with multilineage dysplasia, and with blasts between 20% and 30% [104]. The impact of azacitidine on the survival of MDS patients was further investigated in the international phase III AZA-001 trial. A total of 358 patients with intermediate-2 or high-risk MDS according to the International Prognostic Scoring System (IPSS) were randomized to receive either azacitidine or a conventional care regimen (CCR) that included either low-dose cytarabine, conventional AML induction chemotherapy or supportive care. The results obtained from the phase III randomized AZA-001 trial demonstrated that azacitidine significantly prolonged overall survival (OS) in higher-risk MDS compared with CCR. In fact, patients treated with azacitidine achieved a CR or PR of 29% versus 12%, a two-year OS of 51% versus 26% and a median time to AML transformation or death of 13 months versus 7.6 months when compared with the CCR group. Furthermore, CR was not necessary to prolong OS, since patients with HI had similar two-year survival rates [105]. In March 2009, azacitidine was also approved by the European Medicines Agency for patients who are not eligible for allogeneic hematopoietic stem cell transplantation with the following diagnoses: intermediate- or high-risk MDS according to IPSS, or chronic myelomonocytic leukemia with 10%-29% blasts without myeloproliferative disorder, or patients with AML with 20%- 30% blasts and multilineage dysplasia according to the WHO classification [106]. In a recent large phase III multicenter randomized trial, the AZA-AML-001 study, 488 AML patients aged 65 years or older, with newly-diagnosed or secondary AML with >30% bone marrow blasts and white blood cell counts ≤15 × 10^9^/L were randomized to receive either azacitidine (75 mg/m^2^/day for seven days subcutaneously of each 28-day cycle) or CCR. Interestingly, in the larger group of older AML patients with >30% bone marrow blasts (n = 480), azacitidine prolonged median OS compared with CCR by 3.8 months (10.4 versus 6.5 months) [107].

5-AZA-CdR has been first explored in several phase I/II studies, which showed safety and efficacy and reported encouraging results in AML and MDS patients. These initial data, together with the reported activity of low concentrations of 5-AZA-CdR to induce cellular differentiation *in vitro*, provided the rationale to investigate the activity and toxicity of lower doses of 5-AZA-CdR in further studies (for review see [108, 109, 110). These results led to the first phase III randomized trial of 5-AZA-CdR compared with supportive care in 170 MDS patients, which formed the basis for the FDA approval of decitabine. Patients were randomly assigned to receive either supportive care or decitabine at a dose of 15 mg/m^2^ as a 3-hour infusion every 8 hours for 3 days, repeated every 6 weeks, plus supportive care. 5-AZA-CdR was clinically effective in the treatment of patients with MDS, providing durable responses and improving time to AML transformation or to death, with an observed response rate of 17% {Kantarjian, 2006 #1191]. In a subsequent study, 22 MDS patients who had previously responded to low-dose 5-AZA-CdR were re-treated at the time of disease relapse at a median of 11 months after the last course of initial treatment [111]. Results showed that 45% patients were still responsive, indicating persistent sensitivity to the drug. However, the duration of the second response was inferior to that of the first remission and upfront resistance to the second treatment was also noted, suggesting that a longer period of initial treatment might result in an increased clinical benefit [111]. To address this issue, other 5-AZA-CdR schedules have been evaluated and further improvement was observed with continued treatment. Kantarjian et al. carried out a randomized phase III study, including a 5-day schedule at 20 mg/m^2^ intravenously (IV), a 5-day schedule at 20mg/m^2^ subcutaneously, and a 10-day schedule at 10 mg/m^2^ IV, in 95 patients with MDS and CMML. Each course of decitabine was delivered every 4 weeks and response or failure of therapy were evaluated after at least three courses. The 5-day IV schedule was considered the optimal schedule, since it gave the highest CR rate [112]. Similar results were observed in a phase II study in patients with AML treated with decitabine 20 mg/m^2^ IV for 5 consecutive days of a 4-week cycle [113]. Despite the success of azacitidine and 5-AZA-CdR in the treatment of hematologic malignancies, the *in vivo* mechanisms of these DNMTi still need to be elucidated to guide further trials. The metabolic instability of azacitidine and 5-AZA-CdR has prompted the development of modified analogs, including SGI-110, a dinucleotide that includes a deoxyguanosine and is largely resistant to cytidine deaminase activity [114]. SGI-110 has shown a good tolerability *in vivo* [115] and antitumor activities in xenograft models and in primates [114, 116, 117]. SGI-110 is currently being studied in a randomized phase I-II-III clinical trials focused on treatment of patients with AML and MDS with promising results [118] (Table 1). Several phase I studies with 5-AZA-CdR have also been conducted in NHL patients, but the response to therapy was moderate [119–121]. Other clinical trials are currently testing the ability of DHA to potentiate the activity of conventional chemotherapeutic agents in hematological malignancies.

### IDH inhibitors

Early clinical data obtained with IDH inhibitors suggested their activity in patients with advanced hematologic malignancies, including relapsed/refractory AML and MDS. AG-6780 is a small molecular inhibitor of mutant IDH2 Treatment with AGI-6780 was able to stimulate the expression of maturation markers in leukemic cells *in vitro*, indicating the induction of differentiation [122]. AG-221, another inhibitor of mutant IDH2, has demonstrated selective and sensitive effects on AML cells harboring IDH2 mutations both *in vitro* and *in vivo*. AG-221 has been recently introduced into phase I/II clinical trials for patients with AML and angioimmunoblastic T-cell lymphoma with encouraging preliminary results [123].

### HDAC inhibitors

Given that increased HDAC expression and activity are found is many hematological malignancies, HDAC inhibitors (HDACi) have been extensively used for the treatment of B- and T-cell malignancies (Table 2). Based on their chemical structures, HDACi have been divided into several classes: short-chain fatty acids, hydroxamic acids and hydroxamic acid-based hybrid polar compounds, cyclic tetrapeptides, benzamides and miscellaneous compounds. The antineoplastic activity of these drugs is related to altered gene expression and to changes in non-histone proteins in virtually all most cancer-related pathways. Evidence accumulated so far indicates that the therapeutic potential of HDACi probably stems from their ability to induce selective cell cycle arrest, differentiation and/or apoptosis in different cell types by modulating the expression of target genes [79]. To date, several HDACi have been approved for cancer therapy by the FDA.

**Table 2:** Summary of clinical trials examining Histone Deacetylase, Histone Methyltransferase, and Bromodomain inhibitors in hematological malignancies

HDAC inhibitor	Phase	Combination	Hematological disease	Identifier	State
4SC-202	I		AHM	NCT01344707	Completed
Abexinostat	I		HL, NHL, MM	NCT01149668	Completed
	I/II		HL, FL, MCL	NCT00724984	Completed
Belinostat	I	CHOP	PTCL	NCT01839097	Ongoing
	I		HL, B-cell, T-cell or NK-NHL	NCT00413075	Completed
	II		PTCL	NCT00865969	Completed
	II		MDS	NCT00357162	Completed
	II		MM	NCT00131261	Completed
	II		AML	NCT00357032	Completed
	II		B-cell-NHL	NCT00303953	Completed
	II		CTCL, NHL, PTCL	NCT00274651	Terminated
CI-994	II		MM	NCT00005624	Completed
CUDC-907	I		HL, MM, NHL	NCT01742988	Recruiting
Entinostat	I		HL, NHL	NCT00020579	Completed
	I		ALL, AML, CML, MDS, MM	NCT00015925	Completed
	II		HL	NCT00866333	Ongoing
Givinostat	I/II		HL	NCT00496431	Terminated
	II		CLL	NCT00792831	Terminated
ITF2357	II		MM	NCT00792506	Terminated
Mocetinostat	I/II		DLBCL, FL	NCT02282358	Recruiting
	II		HL	NCT00358982	Terminated
Panobinostat	I	Everolimus	HL, MM, NHL	NCT00962507	Completed
	I		CTCL	NCT00412997	Completed
	I		HL, NHL	NCT01032148	Recruiting
	I		AML	NCT01242774	Completed
	I		HL	NCT00742027	Completed
	I		MM	NCT00532389	Completed
	I/II	Everolimus	HL, MM, NHL	NCT00918333	Ongoing
	I/II		AHM	NCT00621244	Completed
	I/II		AML, MDS	NCT01451268	Recruiting
	II	Rituximab	DLBCL	NCT01282476	Ongoing
	II	Rituximab	DLBCL	NCT01238692	Ongoing
	II		NHL	NCT01261247	Ongoing
	II		NHL	NCT01090973	Terminated
	II		AML	NCT00880269	Completed
	II		NHL	NCT01090973	Terminated
	II		CTCL, Adult T-cell Leukemia/Lymphoma	NCT00699296	Terminated
	II		ALL, AML	NCT00723203	Terminated
	II		MDS	NCT00939159	Terminated
	II		MDS	NCT00594230	Terminated
	II		MM	NCT00445068	Terminated
	II/III		CTCL	NCT00490776	Completed
	II/III		CML	NCT00451035	Completed
	II/III		CML	NCT00449761	Completed
	II/III		CTCL	NCT00425555	Completed
Pivanex	II		CLL	NCT00083473	Terminated
Quisinostat	I		MDS	NCT00676728	Terminated
	II		CTCL	NCT01486277	Ongoing
Resminostat	II		HL	NCT01037478	Completed
Rocilinostat	I/II		HL, NL	NCT02091063	Recruiting
	I/II		MM	NCT01323751	Ongoing
Romidepsin	I/II	Azacitidine	HL, NHL	NCT01998035	Recruiting
	II		CTCL, PTCL	NCT00007345	Ongoing
	II		NHL	NCT00077194	Completed
	II		NHL	NCT00383565	Terminated
SHAPE	II		CTCL	NCT02213861	Recruiting
SHP-141	I		CTCL	NCT01433731	Completed
Tefinostat	I		AML, CLL, CML CMML, HL, MDS, MM, NHL	NCT00820508	Completed
VPA	II		CLL	NCT00524667	Terminated
Valproate	I/II	Rituximab, CHOP	DLBCL	NCT01622439	Recruiting
	I/II		MDS	NCT00776503	Completed
Vorinostat	I		B-cell lymphomas excluding CLL	NCT01276717	Completed
	I		CTCL	NCT00771472	Completed
	I		HL, NHL	NCT00127140	Completed
	I		AML, CLL, CML CMML, HL, MDS, MM, NHL	NCT00005634	Completed
	I	Lenalidomide	HL, NHL	NCT01116154	Terminated
	I/II	Rituximab, CHOP	DLBCL	NCT00972478	Ongoing
	I/II	Cyclophosphamide, Etoposide, Prednisone, Rituximab	DLBCL	NCT00667615	Ongoing
	I/II	Rituximab, Ifosfamide, Carboplatin, Etoposide	MCL, NHL	NCT00601718	Completed
	I/II	CHOP	NHL	NCT00787527	Completed
	I/II		MM	NCT00857324	Terminated
	II	Bortezomib	DLBCL, MCL	NCT00703664	Ongoing
	II		B-NHL, FL, MCL	NCT00875056	Ongoing
	II		NHL	NCT00253630	Ongoing
	II		NHL	NCT00077194	Completed
	II		AML	NCT00305773	Completed
	II		HL	NCT00132028	Completed
	II		CTCL	NCT00958074	Terminated
	II		MDS	NCT00486720	Terminated
**HMT inhibitor**	**Phase**		**Hematological disease**	**Identifier**	**State**
EPZ-5676	I		ALL, AML	NCT02141828	Ongoing
	I		ALL, AML, MDS	NCT01684150	Ongoing
EPZ-6438	I/II		B-cell lymphomas, DLBCL, FL	NCT01897571	
**BET inhibitor**	**Phase**		**Hematological disease**	**Identifier**	**State**
FT-1101	I		AML, MDS	NCT02543879	Recruiting
CPI-0610	I		HL, NHL	NCT01949883	Recruiting
	I		MM	NCT02157636	Recruiting
	I		ALL, AML, CML, MDS	NCT02158858	Recruiting
GSK525762	I/II		AML, NHL, MM	NCT01943851	Recruiting
OTX015	I		AML, ALL, DLBCL, MM	NCT01713582	Ongoing

a AHM, advanced hematologic malignancies; AML, acute myeloid leukemia; BET, bromodomain and extra-terminal motif; CHOP, Cyclophosphamide/Vincristine/Doxorubicin/Prednisone; CLL, chronic lymphocytic leukemia; CML, chronic myeloid leukemia; CMML, chronic myelomonocytic leukemia; CTCL, cutaneous T-cell lymphoma; DLBCL, diffuse large B-cell lymphoma; FL, follicular lymphoma; HDAC, histone deacetylase; HL, Hodgkin's lymphoma; HMT, histone methyltransferase; MCL, mantle cell lymphoma; MDS, myelodysplastic syndrome; MM, multiple myeloma; NHL, non-Hodgkin lymphoma; PTCL, Peripheral T-Cell Lymphoma; VA, valproic acid.

b Identifier of the trial as retrieved April, 2016, from http://clinicaltrials.gov.

#### Short-chain fatty acids

These compounds represent a class of HDACi with simple structures that have shown clinical potential in various studies. Valproic acid (VPA) and phenylbutyrate are two well characterized compounds that belong to this class of compounds. VPA has been increasingly studied in clinical trials as a single agent or in combination therapies. VPA exerts a wide range of effects on AML and results from previous studies have clearly demonstrated that VPA has antiproliferative and pro-apoptotic effects on AML cells [124]. In a phase II study of AML and MDS, 66 patients were initially treated with VPA alone with the later addition of all-*trans*-retinoic acid (ATRA) in non-responsive or relapsed patients. HI was observed in 24% of patients, with a median response duration of 4 months [125]. In a phase I/II study, 44 AML and MDS patients were treated with escalating doses of VPA and concomitantly with a fixed dose of 5-AZA-CdR. Global DNA hypomethylation and histone H3 and H4 acetylation were associated with p15 reactivation in PBMC from patients. However, no correlation was found between these epigenetic modifications and the clinical response. Furthermore, the observed response rate (22%) was much lower than that observed in trials in which 5-AZA-CdR was used alone, thus suggesting that VPA did not offer a significant improvement to the response [126]. VPA has been used in clinical trials for AML and MDS, also in combination with ATRA and azacitidine. In a trial conducted by Soriano et al., 49 AML and 5 MDS patients were treated with a high-dose intermittent schedule of VPA (50 mg/kg for 7 days) combined with the approved azacitidine dosage and ATRA given at 45 mg/m^2^ for 5 days. The combination was safe and clinically active, with an overall response rate of 42%, and 12 (22%) CR. Furthermore, global DNA hypomethylation and histone acetylation were observed but they did not correlate with the clinical response, thus suggesting that responses to HDACi are likely related to non-histone acetylation or to other mechanisms [127]. A further study combining azacitidine (75 mg/m^2^) and VPA (35-50 mg/kg) for 7 days followed by ATRA (45 mg/m^2^) for 21 days was conducted in patients with high-risk AML or MDS. Among the 65 patients enrolled in this study, 14 showed a PR and 3 a CR. Interestingly, promoter demethylation of four genes (FZD9, ALOX12, HPN, and CALCA) was associated with the clinical response [128]. A shortened azacitidine schedule (75 mg/m^2^ for 5 days) plus VPA, ATRA, and theophylline was evaluated in patients with AML or MDS. In 15 out of 79 patients achieving CR, leukemic stem cells were substantially reduced, but never eradicated, since expansion of this population took place before morphological relapse [129], thus suggesting that treatment interruption might be associated with rapid relapse.

#### Hydroxamic acids

Hydroxamic acids include the majority of HDACi currently employed in clinical trials for the treatment of hematological diseases. Among the hydroxamic acids, vorinostat (SAHA, Zolina) was approved by the FDA in 2006 for the treatment of patients with cutaneous T-cell lymphoma (CTCL), a rare type of NHL of the skin. In pivotal phase II clinical trial, the daily treatment of refractory CTCL patients with 400 mg of vorinostat showed a median duration of response of approximately 185 days with a good tolerability and safety profile [130]. Vorinostat treatment was also safe and effective in another phase II clinical trial that involved patients with relapsed/refractory NHL and mantle cell lymphoma [131]. At the molecular level, the antiproliferative effect of vorinostat leads to accumulation of acetylated histones, p21, BAX, STAT6, and caspases, resulting in cell cycle arrest, growth inhibition, apoptosis, and differentiation of cells from AML, MDS, and CTCL patients [132, 133]. Preclinical studies combining rituximab and vorinostat reported increased rituximab activity in B-cell NHL by preventing NF-kB nuclear translocation and promoting its degradation [134]. This combination was further evaluated in a phase II clinical trial in 28 patients with indolent B-cell NHL. The regimen was well-tolerated and appeared promising, with a median progression-free survival (PFS) of 29.2 months for all patients, 18.8 months for relapsed/refractory patients; PFS was not reached in untreated patients [135]. Vorinostat is currently being investigated alone or in combination therapies in several clinical trials for different hematological malignancies (for review, see [136]).

Besides vorinostat, panobinostat (LBH589), belinostat (PDX-101, and pracinostat (SB939), represent a second generation of hydroxamate-based compounds. Panobinostat was recently approved by the FDA for use in combination with bortezomib and dexamethasone in patients with MM. The approval was based on findings in patients with relapsed or relapsed and refractory MM who participated in a clinical trial called PANORAMA1 [137] Panobinostat potently induced cell cycle arrest, apoptosis, and H3K9 and H4K8 hyperacetylation in ALL [138]. Panobinostat was shown to improve the antileukemic effect of fludarabine through a predominantly apoptotic mechanism. The synergism is striking *in vitro* in cell lines, in patient cells, but also in an *in vivo* xenograft model of AML [139]. Furthermore, panobinostat sensitizes leukemic blasts to treatment with cytarabine and daunorubicin by suppressing the expression of BRCA1, CHK1, and RAD51 through transcriptional mechanisms [140]. A phase I/II study combining panobinostat plus decitabine in ederly patients with high risk MDS or AML is currently evaluating the efficacy and safety of this combination [118, 141]. Panobinostat is also under investigation in a phase I/II study in combination with azacitidine in adult patients with MDS, CMML, or AML [118]. Belinostat was approved on 2014 for the treatment of patients with relapsed or refractory peripheral T-cell lymphoma (PTCL) upon results from the phase II BELIEF study [142]. The anticancer effect of belinostat is mediated through the acetylation of H3 and H4, which was seen both *in vivo* and *in vitro* after belinostat exposure. Cancer cell growth inhibition and apoptosis were associated with these increased levels of acetylation [143]. Belinostat is currently being evaluated in phase I/II trials in CTCL and PTCL (for review see [144]), whereas it has shown only minimal activity in AML [145] and no effect in MDS [146]. In DLBCL and PTCL models, belinostat has also been investigated in combination with DNMTi, and synergistic action was observed [147].

Pracinostat gained Orphan Drug status by the FDA in 2014 for future development in AML. In a phase I study of older AML patients, pracinostat induced prolonged CRR of 14% lasting for 206 and 362 days. A phase II trial of pracinostat in combination with azacitidine in patients with high-risk MDS yielded an 89% objective response rate, including 78% CR or CR with insufficient blood count recovery and 56% complete cytogenetic responses. Based on these encouraging results, several studies are currently recruiting patients with MDS or AML [118, 148].

#### Benzamides

Benzamides are synthetic compounds with efficient HDAC inhibitory activity that is mediated by the targeting of the Zn^2+^ ion in the catalytic pocket of the enzyme. [149]. Entinostat (MS-275) and mocetinostat (MGCD0103) are under clinical evaluation as single agents and in combination with other drugs. Entinostat has been also combined with azacitidine in patients with AML and MDS, but the overlapping schedule of this combination was not superior to azacitidine monotherapy and was associated with pharmacodynamic antagonism [150]. In preclinical studies in HL, entinostat increased H3 acetylation, up-regulated p21 expression, and promoted the intrinsic apoptosis pathway by downregulating Bcl-2 and Bcl-xL proteins [151]. In *in vivo* experiments, entinostat was also found to enhance the antitumor activity of rituximab-sensitive and -resistant B-cell lymphoma cell lines [152].

#### Cyclic peptides

Several cyclic peptides isolated from microorganisms have been described to possess HDAC inhibitory activity. Romidepsin (Istodax, FK228, FR901228, depsipeptide), a natural product obtained from the bacterium *Chromobacterium violaceum*, was approved by the FDA in 2009 for the treatment of the refractory form of CTCL. The FDA approval of romidepsin for the treatment of CTCL was based on two large phase II studies: a multi-institutional study and an international study including 71 and 96 patients, respectively. The treatment schedule was identical and produced overall response rates of 34-35%, with a CR in about 6% of patients in both studies. The median duration of response was 13.7 months [153]. In 2011, romidepsin was also approved for PTCL in patients who had received at least one prior therapy. In the pivotal study, including 130 patients with relapsed or refractory PTCL, objective responses were seen in 25% and CR in 15% [154]. The median duration of response was 28 months with a median follow-up of 22.3 months [155]. In patients with CLL, AML and MDS, romidepsin did not show significant clinical efficacy, but exhibited some biological activity as indicated by an increase in p21 protein expression concurrent with H4 acetylation of the p21 promoter gene [156, 157]. Recently, it has been demonstrated that romidepsin also has DNA hypomethylating activity, which might inhibit the Wnt/β-catenin pathway in T-lymphoblastic cells [158]. Romidepsin is currently being evaluated in several studies, either as a single agent or in combination with other drugs for treating mainly T-cell lymphoma.

### HAT inhibitors

Unlike HDACs, little is known about inhibitors of HATs (HATi) in B- or T-cell cancers, and no HATi are currently approved by the FDA. HATi may have therapeutic potential and several natural HATi are under evaluation for their anti-cancer properties. Among these natural compounds, curcumin has gained increased attention as a potential anti-cancer drug and has being investigated in a variety of tumors, including MM [159]. Curcumin has been shown to possesses intrinsic HATi activity specific for p300/CBP both *in vitro* and *in vivo* and induce cell proliferation arrest and apoptosis [160]. However, curcumin has many protein targets, and thus it is not clear as to whether its effects are only due to its anti-HAT activity [161]. Anacardic acid, a chemical compounds found in the shell of the cashew nut, is a potent inhibitor of several HAT, including p300, PCAF, and TIP60. Anacardic acid was demonstrated to inhibit the growth of Jurkat T-cell leukemia cells [162], which express two TIP60 variants, including one with a deleted HAT domain [163].

### HMT inhibitors

So far, only a few HMT inhibitors (HMTi) are known, and most of them were discovered through random screening approaches. Deazaneplanocin A (DZNep) is an HMTi that has a wide range of anticancer effects in several human cancers. In particular, DZNep has been found to deplete EZH2 levels and to inhibit H3K27 trimethylation in AML cell lines in a dose-dependent manner. DZNep treatment increases both mRNA and protein levels of p16, p21, and p27 in AML cells, causing cell cycle arrest and apoptosis. Co-treatment with panobinostat enhances the antileukemic effects of DZNep and was found to synergistically improve the survival of mice implanted with AML cells [164]. Two other EZH2 inhibitors, GSK126 and EPZ005687, have shown preferential effectiveness in suppressing the growth of lymphoma-associated mutants of EZH2 in comparison to those with wild-type EZH2 [165, 166]. In a study by McCabe et al, GSK126 inhibited the proliferation of Y641 EZH2 DLBCL cell lines and induced a 50% loss of H3K27 trimethylation. Besides cell-based studies, treatment of xenografted DLBCL models resulted in either tumor regression or tumor inhibition, depending on the dosing regimen, and GSK126 was well tolerated [165]. Similarly, EPZ0056687 has been shown to induce apoptotic cell killing in heterozygous mutant EZH2 Y641 or alanine 677 lymphoma cells, with minimal effects on wild-type cell proliferation [166]. EPZ004777 was the first specific DOT1L small molecular inhibitor to be been extensively characterized. Daigle et al. reported decreased H3K79 methylation levels and selective inhibition of leukemogenic genes expression in MLL cells exposed to EPZ004777. Furthermore, administration of EPZ004777 resulted in selective apoptosis of cell lines harboring MLL gene translocation, with minimum effect on non-MLL-rearranged cells. Finally, *in vivo* EPZ004777 treatment increased overall survival in an MLL xenograft model [167]. EPZ-5676 is another potent and selective DOT1L inhibitor which selectively kills cells containing the MLL chromosomal translocation, whereas it shows little effect on leukemia cells that lack this translocation. In a rat xenograft model of MLL-rearranged leukemia, EPZ- 5676 was able to induce significant cancer growth inhibition. Furthermore, complete regression was achieved following 21 days of continuous intravenous administration of EPZ-5676 and tumor regrowth was not observed until the end of the experiment [168]. EPZ-5676 has now advanced to phase I clinical trials in patients with advanced hematological malignancies, including AML with MLL fusions (Table 2).

### BET inhibitors

In 2010, two independent research groups demonstrated the excellent activity of two BET proteins small-molecule inhibitors, known as I-BET762 (GSK 525762A) and JQ1 [169, 170]. During a screening that evaluated the ability of synthetic compounds to bind selectively to individual proteins in cell lysates, I-BET762 showed the highest affinity interaction with the acetyl-lysine binding site of BRD4-BD1. In this study, I-BET762 significantly improved the survival of C57BL/6 mice with severe sepsis by disrupting chromatin complexes responsible for the expression of crucial inflammatory genes [169]. The inhibitor JQ1 was initially shown to have efficacy in the context of nuclear protein in testis (NUT) midline carcinoma (NMC). In the majority of NMC the two N-terminal BET bromodomains of BRD4 are fused with NUT via the t(15;19) translocation to create an oncogenic fusion gene whose product is driven by the BRD4 promoter. Treatment of NMC cell lines and xenograft models with JQ1 displaced the BRD4 fusion protein from nuclear chromatin and induced squamous differentiation, thus exhibiting specific anti-proliferative effects [170]. The results of I-BET762 and JQ1 treatments have encouraged the development of other BET inhibitors with clear antiproliferative and pro-apoptotic effects in several hematological malignancies, including MM [171], NHL [172], AML [95, 173] and ALL [174]. Based on the observed down-regulation of a common transcriptional program that includes critical oncogenic targets such as BCL2 and c-MYC, several phase I clinical trials have been initiated to explore the efficacy of BET inhibitors in AML and other hematological malignancies (Table 2). The first published evidence of the clinical activity of BET inhibitors refers to a phase I clinical trial with OTX015, in which promising antitumor activity was seen in both acute leukemia and other hematological malignancies (Patrice Herait, AACR Annual Meeting, San Diego, LA, USA; Oral communication, April, 2014).

## CONCLUSIONS

Somatic alterations in genes involved in DNA methylation and histone modifications have emerged central events in the development and progression of hematological malignancies. Loss or gain of function mutations affecting catalytic domains deregulate the activity of epigenetic enzymes and may promote leukemogenesis by altering the normal self-renewal and differentiation of HSC. Furthermore, fusion proteins generated by translocation often mediate their oncogenic potential by directly or indirectly interfering with epigenetic modifying activities. However, the epigenetic mechanisms exploited by leukemic fusion proteins to drive the induction and maintenance of the leukemic state are still far from being elucidated. So far, DHA and HDACi have been widely used to evaluate the potential of epigenetic modulation to benefit patient outcome. Nevertheless, the currently available epigenetic drugs are non-specific, so new therapeutic molecules with well-defined targeting of the epigenetic machinery should be developed. Rational combination of available epigenetic drugs with targeted therapies also warrants investigation in the clinical setting. Further characterization of the genetics of hematological malignancies, together with a better understanding of the role of epigenetic alterations in leukemic transformation will probably provide amenable targets for next-generation epigenetic drugs.
